# A custom ddPCR method for the detection of copy number variations in the nebulin triplicate region

**DOI:** 10.1371/journal.pone.0267793

**Published:** 2022-05-16

**Authors:** Lydia Sagath, Vilma-Lotta Lehtokari, Carina Wallgren-Pettersson, Katarina Pelin, Kirsi Kiiski

**Affiliations:** 1 Folkhälsan Research Center, Helsinki, Finland; 2 Department of Medical Genetics, Medicum, University of Helsinki, Helsinki, Finland; 3 Molecular and Integrative Biosciences Research Programme, Faculty of Biological and Environmental Sciences, University of Helsinki, Helsinki, Finland; Universidade Lisboa, Instituto superior Técnico, PORTUGAL

## Abstract

The human genome contains repetitive regions, such as segmental duplications, known to be prone to copy number variation. Segmental duplications are highly identical and homologous sequences, posing a specific challenge for most mutation detection methods. The giant nebulin gene is expressed in skeletal muscle. It harbors a large segmental duplication region composed of eight exons repeated three times, the so-called triplicate region. Mutations in nebulin are known to cause nemaline myopathy and other congenital myopathies. Using our custom targeted Comparative Genomic Hybridization arrays, we have previously shown that copy number variations in the nebulin triplicate region are pathogenic when the copy number of the segmental duplication block deviates two or more copies from the normal number, which is three per allele. To complement our Comparative Genomic Hybridization arrays, we have established a custom Droplet Digital PCR method for the detection of copy number variations within the nebulin triplicate region. The custom Droplet Digital PCR assays allow sensitive, rapid, high-throughput, and cost-effective detection of copy number variations within this region and is ready for implementation a screening method for disease-causing copy number variations of the nebulin triplicate region. We suggest that Droplet Digital PCR may also be used in the study and diagnostics of other segmental duplication regions of the genome.

## 1. Introduction

Approximately 5% of the human genome is composed of segmental duplications (SD)—sequences of 10–300kb in size, repeated at least two times in the genome. Repetitive regions of the genome are particularly prone to copy number variations (CNVs) [1–4].

The giant sarcomeric gene nebulin (*NEB*, MIM ID *161650) is located in the chromosomal region 2q23.3. It spans over 249 kb of genomic region and has 183 exons. Mutations in *NEB* are known to cause nemaline myopathy (NM; MIM IDs: NEM2 #256030) and other congenital myopathies. *NEB*-caused NM is inherited mainly in an autosomal recessive fashion, but lately, rare dominant mutations in the form of large deletions have also been described [5, 6]

In its mid-region, *NEB* harbors a 30 kb SD where eight exons are repeated three times (exons 82–89, 90–97, 98–105). Each 8-exon-block spans 10 kb of genomic region, and the repeats are highly similar between themselves [7]. The ratio of the 8-exon-block compared with the diploid genome is thus 3:1, and the block has been named the *NEB* TRI region. This region has been shown to harbor normal as well as pathogenic copy number variation. It has been shown that a single allele tolerates the gain or loss of one but not several repeated blocks [8].

Array Comparative Genomic Hybridization (aCGH) still constitutes the gold standard method for CNV analysis, although massively parallel sequencing-based methods are continuously being developed and are rapidly improving in both accuracy and reliability [9]. In CNV detection, SD regions remain a challenge for both aCGH and sequencing-based methods because of their repetitive nature. When composing arrays, it is usually impossible to design unique probes to target these regions, which are often left without probes. Furthermore, their repetitive nature tests both amplification steps and alignment in sequencing-based methods.

In an attempt to investigate both the *NEB* TRI region and a similar, yet shorter, repetitive region in another sarcomeric giant, titin (*TTN*, MIM ID *188840), we have previously designed and published validated custom tiling array designs for the detection of CNVs in nemaline myopathy and other neuromuscular disorders. These include the SD regions also (the NM- and NMD-CGH-arrays) [10, 11]. To date, running more than 300 DNA samples from families with persons affected by neuromuscular disorders on the NM- and NMD-arrays, we have identified CNVs in the *NEB* TRI region in altogether 13%. In a third of these, the CNVs have been interpreted as being pathogenic.

Although the custom arrays yield a high sensitivity in SD CNV detection, very high copy numbers (CN) of the *NEB* TRI region are difficult to determine precisely due to the log2-based mathematical model commonly used in aCGH data analysis [12]. This situation may be compared with attempting to estimate the grade of mosaicism of a CNV in a sample. The aCGH method has indeed been used in such studies, even though this is not its original intended use [6, 13, 14].

Furthermore, the custom arrays come at a relatively high cost per sample and a turn-around time of roughly three days. The optimal DNA requirements are high: 1,000 ng of good-quality genomic DNA at a concentration of more than 55 ng/*μ*l is needed per sample per run. Especially in cases where one myopathy-causing mutation in *NEB* has been identified, and a CNV in the *NEB* TRI region is suspected to be the other causative mutation, running a custom CGH-array is excessively expensive and time-consuming. Most importantly, setting up a custom ddPCR assay is reasonably straightforward, especially if the required apparatus is already available—which is more likely, as ddPCR is more versatile a method than array-CGH.

Droplet Digital PCR (ddPCR) allows for the precise quantification of specific amplified nucleic acid molecules. Each reaction is partitioned into a theoretical optimum of 20,000 droplets, allowing for independent amplification of target molecules. When combined with target-specific fluorochrome-labeled probes, the fluorescence of each droplet can be read and quantified, and rare DNA target copies can be detected with high sensitivity. Using Poisson statistics and a reference gene of known ploidy, the ratio between target molecules and reference molecules allows for the determination of the CN of the target nucleic acid molecule.

The ddPCR method is helpful for a multitude of purposes, e.g., gene expression studies, mutation and gene edit detection, residual DNA detection, and CNV detection. Digital PCR systems are available from several manufacturers, but the principle of the method remains the same. Designing custom ddPCR assays or acquiring validated assays is easier and more cost-effective than designing or gaining access to custom CGH-arrays. Furthermore, both the running cost and the DNA mass requirement of one sample is roughly 1/100 of a sample run on a custom CGH-array, and the turn-around-time is cut to one-third.

Within the context of neuromuscular disorders, ddPCR has been used in only a few studies. In spinal muscular atrophy patients, the method has been used to determine *SMN1* and *SMN2* exon 7 copies [15]. In addition, while it appears not to have been used for CN determination of dystrophin in Duchenne muscular dystrophy patients, it has been used in the quantification of exon skipping in the development of antisense oligonucleotide therapy [16]. Furthermore, it has been used in CNV analysis of the BRCA1 gene in high-grade serous ovarian cancer tissue samples and presented as a promising method for diagnostics [17]. To our knowledge, no papers have been published reporting CNV analysis of intragenic segmental duplication regions using ddPCR.

Here, we present our *NEB* TRI targeted ddPCR-based screening method as a precise, sensitive, high-throughput, and cost-effective method for the CNV analysis of the *NEB* TRI region and as an example of how ddPCR can be used as an analysis tool for the CN determination of SD regions.

## 2. Materials and methods

### 2.1. Samples

Altogether 130 DNA samples were acquired for this study. Of these, 26 were healthy controls from the Fondation Jean Dausset-CEPH, and 20 were healthy controls from the Finnish Red Cross Blood Service. In addition, we used 84 DNA samples from our sample collection of congenital myopathy patients and their family members. The *NEB* TRI CN of all samples had previously been determined using the NM- or NMD-arrays [10, 11].

The study was approved (approval number 6/E7/05) by the Ethics Committee of the Children’s Hospital, University of Helsinki, Finland. The approval was renewed by the Ethics Review Board of Helsinki University Hospital in 2021. Samples were obtained according to the Declaration of Helsinki of 1975, and written consent was obtained from subjects, or data were analyzed anonymously where appropriate.

The patient samples used were heterogeneous in terms of clinical diagnosis, ethnic background, and age. Furthermore, some DNA samples had been received extracted while some were extracted in our laboratory. The DNA stocks were extracted from leukocytes or saliva, eluted into EDTA, TE-buffer, or water, and stored at -20°C or -80°C. The DNA concentration and quality were measured with DeNovix DS-11 FX+ Spectrophotometer/Fluorometer (DeNovix Inc., Wilmington, DE, USA). Subsequent dilutions for the ddPCR reactions were done in sterile water in appropriate series.

### 2.2. Microarray design, protocol, and data analysis

All samples had previously been run on the NM-CGH-array or the NMD-CGH-array as previously described [10, 11].

To avoid any subtle differences caused by the genome-wide normalization of the analysis software (CytoSure Interpret Software v.4.11.30, Oxford Gene Technology Ltd), the previously acquired aCGH data for *NEB* was further manually aligned to gain a baseline of zero. The log_2_ value for the *NEB* TRI region and large portions of the *NEB* gene on either side of the *NEB* TRI were extracted from the aCGH data. The breakpoints used for normalization were Chr2:(152340824_152341131)_(152432955_152433349) and Chr2:(152465657_152465735)_(152592449_152583346). The *NEB* TRI region used to calculate the normalized CN was Chr2:(152433598_152434494)_(152465223_152465448). The genomic locations are given in the reference genome Hg19/GRCh37. The normalized log_2_ value of the *NEB* TRI region was acquired by subtracting the averaged background log_2_ value from the *NEB* TRI region. This value was then used to calculate the estimated CN by assuming a normal CN of 6 and compared with the previously published log_2_ values for different *NEB* TRI CNs (Table 1 in [8]).

### 2.3. Digital droplet PCR

The ddPCR assays were designed, performed, and analyzed according to the updated dMIQE guidelines [18, 19]. The dMIQE checklist is available in S1 Table.

#### 2.3.1. Primer and probe design

**Fig 1:**
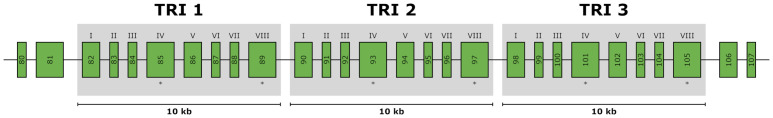
A schematic of the exons in the *NEB* TRI region. The exons targeted by the ddPCR assays are marked with an asterisk (*).

Two custom assays to target two different exons of the eight-exon-block of the *NEB* TRI were designed. The first assay targets the fourth exon of the *NEB* TRI region, corresponding to *NEB* exons 85/93/101, referred to from now on as exon IV. The second assay targets the last exon of the *NEB* TRI region, corresponding to *NEB* exons 89/97/105, referred to from now on as exon VIII (Fig 1). The target selection was limited by unique sequences, sequence GC% and BsuRI restriction site locations.

Primers and probes for the assays were designed using Primer3Plus (https://www.bioinformatics.nl/cgi-bin/primer3plus/primer3plus.cgi/) as per the manufacturer’s suggestions. The primers designed had a melting temperature (Tm) of 55.8–57.5°C (calculated by the nearest-neighbor method), a primer concentration of 300 nM, and a salt concentration of 50 nM), a GC content of 50%, and a length of 20 bp. The amplicons were not allowed to contain the BsuRI cut site sequence GGCC. Amplicon lengths were 104 and 169 bp for exons IV and VIII, respectively.

Hydrolysis probes were designed to have a Tm of approximately 65°C and a GC content of 55–57%. Custom probes were labeled with fluorescein amitide (FAM).

The specificity of primers and probes were verified by the Standard Nucleotide BLAST blastn suite (https://blast.ncbi.nlm.nih.gov/Blast.cgi), allowing three hits for both assays. A commercial PrimePCR ddPCR Copy Number Assay for human *EIF2C1* labeled with hexachloro-fluorescein (HEX) (Cat. No. 10031243, Bio-Rad Laboratories Inc., Hercules, CA, USA) was used as a reference. *EIF2C1* is a diploid gene located on 1p34.3, and also known as Argonaute 1 (*AGO1*).

The probes were manufactured by Bio-Rad Laboratories Inc. All primer and probe sequences along with amplicon lengths are presented in S2 Table. The custom assays were ordered at a primer-probe ratio of 3.6:1 per assay.

#### 2.3.2. Assay optimization

The optimal melting temperature for the assays was determined by replacing the annealing step in the ddPCR cycling program with a thermal gradient (temperatures 55.2°C, 58.0°C, 60.0°C, 60.5°C and 62.3°C) for 1 minute. A melting temperature of 60.0°C was chosen, as it allowed equally good separation between droplet clusters in both assays.

The quantity of DNA was scaled down to 10 ng/reaction as per the manufacturer’s suggestion.

#### 2.3.3. No-template controls

In all runs, individual no-template controls (NTCs) with water were included for both assays in each run. The NTCs were placed in the last wells of each run plate.

#### 2.3.4. Assay protocol

For each well, a 20 *μ*l reaction was prepared, containing 10 *μ*l 2x Bio-Rad ddPCR Supermix for Probes (No dUTP), 1 *μ*l 20x custom assay, 1 *μ*l PrimePCR ddPCR Copy Number Assay for *EIF2C1*, 1 *μ*l BsuRI (Thermo Scientific, Waltham, MA, USA) diluted 1:1 in Fast Digest buffer (Thermo Scientific), and 10 ng of DNA template diluted in 7 *μ*l of sterile water. The final concentration of primers and probes was 900 nM and 450 nM, respectively.

For droplet generation, 20 *μ*l of reaction mixture and 70 *μ*l of Bio-Rad Droplet Generation Oil for Probes were pipetted onto a DG8 cartridge and covered by a DG8 gasket in a DG8 Cartridge Holder. Droplets were generated using the Droplet Generator QX200 (Bio-Rad Laboratories Inc). The droplets were then transferred to ddPCR 96-well plates (Bio-Rad Laboratories Inc.) and sealed with the PX1 PCR Plate Sealer (Bio-Rad Laboratories Inc.). The PCR reaction was performed using the DNA Engine Tetrad 2 Thermal Cycler (Bio-Rad Laboratories Inc) with the ramp rate set to 2°C/sec. The samples were cycled as follows: 95°C 10 minutes; 40 cycles of 94°C 30 seconds, 60°C 1 minute; 98°C 10 minutes; 4°C hold. On each plate, a no-template control for each assay was included. Droplet fluorescence was measured using the QX200 Droplet Reader (Bio-Rad Laboratories Inc) and the QuantaSoft Analysis v.1.7.4.0917 software.

All reagent and consumable manufacturer information and catalog numbers are presented in S3 Table.

### 2.4. Data analysis

#### 2.4.1. Analysis of raw data

The samples were primarily analyzed on the QuantaSoft Analysis v. 1.7.4.0917 and QuantaSoft Analysis Pro v. 1.0.596 (Bio-Rad Laboratories Inc.) softwares.

Intensity thresholds for droplets were set manually per plate, assay, and channel. Each droplet was assigned to a group based on their intensities of the two detection channels: FAM positive-HEX positive, FAM positive-HEX negative, FAM negative-HEX positive, and FAM negative-HEX negative. Assuming Poisson distribution, a ratio between the target and reference channel was automatically calculated by the software. The *NEB* TRI CN was estimated by doubling the thus estimated ratio of assay target to diploid reference.

#### 2.4.2. Filtering of raw data

Droplet data were extracted from the QuantaSoft Analysis software and imported into Microsoft Excel.

The data were filtered by removing runs in which any single droplet cluster had 100 or fewer accepted droplets and runs in which the total accepted droplet count was 10,000 or less. Runs on whole-genome amplified DNA (n = 4), samples from patients with known mosaicism in *NEB* (n = 1), samples with other CNVs in *NEB* (n = 9), samples run successfully in only one of the assays (n = 15), and samples with no successful runs in either assay were excluded. After the filtering steps, results from 98 independent samples remained, with 176 and 162 data rows for *NEB* IV and *NEB* VIII, respectively. Of these 98 samples, 39 were controls and 59 were from neuromuscular disorder patients and healthy family members thereof. The filtered data were extracted as comma-separated value (CSV) files. Subsequent statistical analyses were performed in RStudio v.1.3.959.

#### 2.4.3. Assessment of intra-assay and inter-assay variability

Intra-assay analyses were conducted to assess reproducibility within experiments. The analyses were performed separately for the two custom ddPCR assays (*NEB* IV n = 8, *NEB* VIII n = 9) using duplicates run within the same experiment.

Inter-assay analyses were conducted to assess repeatability between experiments. The analyses were performed separately for the two custom ddPCR assays (*NEB* IV n = 60, *NEB* VIII n = 42). In cases where a sample had been run more than twice in separate experiments or had an intra-assay duplicate, we used the two runs with the highest accepted droplet counts from two separate experiments.

#### 2.4.4. Statistical analysis

To determine the concordance between the ddPCR and aCGH results, we used linear regression analysis, the Pearson correlation coefficient, and a weighted *κ*-analysis [20]. Unweighted *κ*-analysis was used to estimate the level at which the ddPCR assays were able to distinguish between pathogenic and benign CNVs. Linear regression analysis and the Pearson correlation coefficient were also used to evaluate the concordance between the two ddPCR assays.

For the *κ*-analysis, we classified the degree of agreement as 0.81–1.0, almost perfect agreement; 0.61–0.80, substantial agreement; 0.41–0.60, moderate agreement; 0.21–0.40, fair agreement; and <0.20, slight agreement.

The weighted *κ*-analysis was performed for both assays for the concordance between the aCGH and ddPCR results using the assigned aCGH CN and the ddPCR CN estimate rounded up or down to the nearest integer. The range, mean, standard deviation (*σ*), and coefficient of variation in percent (%CV) were extracted and calculated for each CN group for aCGH and the two ddPCR assays separately.

Statistical analysis was executed in RStudio v.1.3.959 using R v.4.0.4 and Microsoft Excel.

## 3. Results

### 3.1. Data overview

Custom ddPCR assays were run on n = 130 samples. After filtering, 98 samples remained for analysis. In these samples, the *NEB* TRI CN ranged from 5 to 14, lacking samples representing CNs of 12 and 13 due to unavailability. The sample numbers per *NEB* TRI CN are presented in Table 1.

**Table 1:** Number and percentage of samples representing different *NEB* TRI CNs. The *NEB* TRI CN of 6 is considered the normal CN, with 5 and 7 being benign CNVs. A deviation of two or more *NEB* TRI blocks is considered a pathogenic CNV.

	Copy number	Number of samples	% of samples
Benign (n = 83)	5	22	22.4
	6	44	44.9
	7	12	12.2
Pathogenic (n = 20)	8	3	3.1
	9	5	5.1
	10	7	7.1
	11	2	2.0
	14	3	3.1
	Total	98	100

Example 2D droplet plots from successful samples and NTCs are provided in S1 Fig.

The mean accepted droplet count was 12,974 (*σ* 1,701.2, %CV 13.1) for the *NEB* TRI exon IV assay and 13,285 (*σ* 1,434.9, %CV 10.8) for the *NEB* TRI exon VIII assay, indicating a reasonably consistent quality in the reaction set up. The droplet counts for both clusters and all accepted droplets are presented in S4 Table. The mean, *σ*, and %CV values for copies per partition are presented in S5 Table.

**Fig 2:**
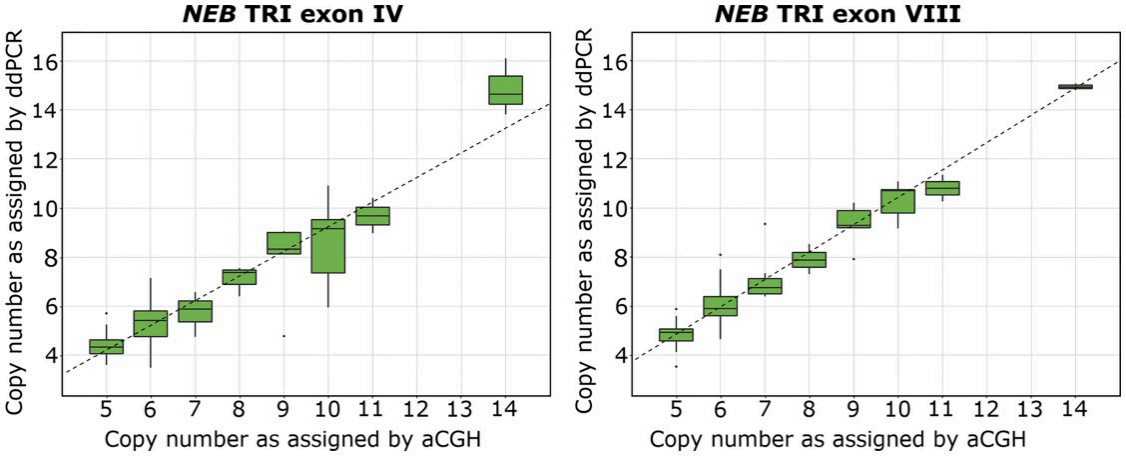
Boxplots visualizing the CN of the *NEB* TRI exon IV and VIII assays in relation to the CN assigned by aCGH with linear regression trend line. The *NEB* TRI exon IV assay gives a relationship with a lower linear slope than the *NEB* TRI exon VIII assay, which seems to follow a 1:1 linear relationship adequately. The dashed lines represent the linear regression trend lines.

The estimated ddPCR CNs are shown plotted against the aCGH-determined *NEB* TRI CN in Fig 2.

The minimum, maximum, and mean values for the *NEB* TRI CN given by the aCGH results and the two ddPCR assays are presented in S6 Table. The average %CV for the NM- and NMD-CGH-arrays was 2.86. For the ddPCR assays, the %CV was 12.14 and 9.22 for the *NEB* TRI exon IV and *NEB* TRI exon VIII assays, respectively.

### 3.2. Intra-assay analysis

The intra-assay duplicate means, standard deviations, and %CV were calculated, along with an average %CV for the complete assay to assess reproducibility. The intra-assay %CV mean for *NEB* TRI exon IV (n = 8) was 3.8, and the intra-assay %CV mean for *NEB* TRI exon VIII (n = 9) was 4.1. The intra-assay analysis is presented in S7 Table.

### 3.3. Inter-assay analysis

The inter-assay duplicate means, standard deviations, and %CV was calculated, along with an average %CV for the complete assay to assess repeatability. The inter-assay %CV mean for *NEB* TRI exon IV (n = 60) was 19.3, and the inter-assay %CV mean for *NEB* TRI exon VIII (n = 42) was 5.1. The inter-assay analysis is presented in S8 Table.

### 3.4. Pearson correlation coefficient

The Pearson coefficient for the *NEB* TRI exon IV assay against the aCGH-determined CN was 0.898. The corresponding Pearson coefficient for the *NEB* TRI exon VIII assay was 0.957. Additionally, we calculated a Pearson coefficient to compare the two ddPCR assays with each other, yielding a value of 0.910.

### 3.5. Linear regression

The linear regression model for the *NEB* TRI exon IV assay yielded an estimate of 1.004, a multiple R^2^ value of 0.807, and an adjusted R^2^ value of 0.805 (p < 0.0001). The linear regression model for *NEB* TRI exon VIII yielded an estimate of 1.115, a multiple R^2^ value of 0.917, and an adjusted R^2^ value of 0.916 (p < 0.0001). The complete linear regression analysis results are presented in S9 Table.

### 3.6. Kappa analysis for copy number variation detection comparison

The *κ*-value for the *NEB* TRI exon IV assay in detection of CN was 0.558, indicating moderate agreement (p < 0.00001) between aCGH and the *NEB* TRI exon IV assay. The *κ*-value for the *NEB* exon VIII assay in detection of CN was 0.778, indicating substantial agreement (p < 0.00001) between aCGH and the *NEB* TRI exon VIII assay. The Kappa tables are available in S10 Table.

### 3.7. Kappa analysis for detection of pathogenicity comparison

The *κ*-value for the *NEB* TRI exon IV assay in detection of pathogenicity was 0.388, indicating a fair agreement (p < 0.0005) between aCGH and the *NEB* TRI exon IV assay. The *κ*-value for the *NEB* TRI exon VIII assay in detection of pathogenicity was 0.774, indicating a substantial agreement (p < 0.00001). The Kappa tables are available in Table 2, with additional statistics in S11 Table.

**Table 2:** The Kappa tables for detection of pathogenicity of assays *NEB* TRI exon IV and exon VIII.

*NEB* TRI exon IV				
		aCGH		
		Benign	Pathogenic	
ddPCR	Benign	58	6	
	Pathogenic	20	14	
	Total	78	20	98
*NEB* TRI exon VIII				
		aCGH		
		Benign	Pathogenic	
ddPCR	Benign	71	1	
	Pathogenic	7	19	
	Total	78	20	98

## 4. Discussion

Our results show that the ddPCR method is a viable option for the detection of CNVs of the *NEB* TRI region. To date, only the NMD-CGH-array and other custom CGH-arrays covering the regions have been reliable methods in CNV analysis of the region. Methods based on algorithms applied to massively parallel sequencing data may detect these variations, but exact CN determination cannot be done reliably. The aim of the study was to develop a method complementary to aCGH to be used for *NEB* TRI CNV screening because, in comparison, the running of a custom-aCGH is both rather costly and time-consuming. As ddPCR is more approachable than aCGH, our custom method allows for anyone with the necessary equipment to adopt the method for *NEB* TRI CNV screening.

Critical evaluation of the data showed that extreme outliers passing the droplet number cutoffs in the analysis pipeline all had reasonable explanations. Among these were factors such as known mosaicism for a nebulin deletion, the sample having been whole-genome amplified, or the DNA otherwise being of low quality, all reflected in the corresponding aCGH data also.

Our data demonstrate the importance of verification in the development of new ddPCR assays. Although our two assays were designed to function in the same PCR conditions, there was a substantial difference between them in terms of accuracy. The *NEB* TRI exon IV assay consistently gave lower CN estimates than expected, while the *NEB* TRI exon VIII assay gave good estimates and showed substantial agreement with aCGH results, both in terms of CN determination and identification of pathogenic CNs. Hence, we believe our assay for *NEB* TRI exon VIII is representative. We appreciate the fact that the designed assays only cover representative portions of the *NEB* SD region and that multiple assays throughout the region would increase their accuracy. However, the design of suitable assays is limited by amplicon size and the repetitive nature of the region.

Based on our experience, different DNA extraction methods, the quality of the DNA, and possible inhibitors of the amplification reaction likely affect ddPCR assays. Access lacking to extensive sample collections of DNA extracted locally and stored identically, it is difficult to draw any conclusions as to which factors may affect the results marginally and which may affect it significantly. This further emphasizes the importance of correct assay optimization and adherence to the dMIQE guidelines [18, 19] to the highest possible degree of reproducibility and repeatability.

Furthermore, manual handling such as pipetting differences between performers may also affect the result. Full automation of the ddPCR assay procedure would be necessary to implement the SD-specific method in a diagnostic setting and would most likely improve both repeatability and reproducibility. The ddPCR method is already being implemented in a diagnostic setting, and these automated ddPCR systems are finding their way into service laboratories. We believe that the usage of ddPCR in various types of diagnostics will eventually replace some of the methods currently in use; one such example is ddPCR replacing MLPA in the detection of single-exon deletions of *BRCA1* [17].

One of the benefits of ddPCR compared with aCGH is the usage of an internal reference gene instead of a complete reference genome. Selecting a reference genome for aCGH to suit the needs for the CNV analysis of regions in which benign CNVs are common, such as the *NEB* TRI region, warrants carefulness. Since aCGH is a genome-wide comparative method, a reference with a known CN of any region of interest is always needed for drawing conclusions, and the task becomes even more challenging if one is interested in more than one such region. For this specific problem, ddPCR offers a more clear-cut solution: the CN is determined by comparing the repetitive region with an intragenomic diploid region.

To our knowledge, and based on our aCGH data, no cases in which the *NEB* SD harbors a CNV of partial repetitive blocks have been recorded. We have, however, identified CNVs spanning one *NEB* TRI block and adjacent regions of the SD [5, 21]. In addition, we have identified one case of a large mosaic CNV spanning the entire SD region in addition to both upstream and downstream flanking regions, resulting in a short transcript [6]. The ddPCR method presented here is thus intended as a first-tier screening method—any findings should be investigated by another validated method, such as the NM- or NMD-CGH arrays (or equivalent), to exclude the possibility of a larger CNV extending beyond the *NEB* TRI region. The last introns of each of the *NEB* TRI region blocks contain *Alu* and LINE repeats, known to contribute to non-allelic homologous recombination. The hypothesis is that these repeats initially contributed to the emergence of the *NEB* TRI region in humans [22] and that they, at the same time, contribute to the susceptibility of the region for CNVs. The relatively high proportion of CNVs observed in the *NEB* TRI region would implicate that these repetitive elements and their intricate replication contribute to the high prevalence of aberrations in *NEB*. The knowledge of CNVs being prevalent in the *NEB* TRI region warrants a search for their presence in other intragenic SDs.

The size of nebulin has been shown to correlate with thin filament and sarcomere lengths, and there seem to be limits for optimal nebulin size within any given species [23]. According to the Ruler Hypothesis [24, 25], large enough gains in CN in the *NEB* TRI region may be pathogenic. We have shown that gains of two or more blocks of *NEB* TRI in one allele segregate with disease and are absent in control samples; therefore, such gains are considered pathogenic [8]. A gain or loss of two or more blocks would significantly lengthen or shorten the nebulin polypeptide, which would impair optimal and energy-efficient force production [23]. While our custom CGH-arrays effectively determine the CN in the *NEB* TRI region, they are comparatively expensive and laborious compared with the now established ddPCR assay. CNV detection algorithms applied to massively parallel sequencing data can sensitively detect variation in SD regions. However, they may not precisely and effectively determine the exact CN of the repeated blocks. In the case of the *NEB* TRI, exact CN determination is crucial, as a one copy gain is considered benign, but a two-copy gain pathogenic.

Our custom ddPCR assays can be used to screen large sample numbers for *NEB* TRI CNVs, especially in samples with one identified mutation in *NEB* and samples from families in which a *NEB* TRI CNV has been found. We also suggest that the method may be applicable to other segmental duplications and similar intragenic SDs regions of the genome and approach the subject in our ongoing research.

## Supporting information

S1 Table dMIQE checklist. (XLSX)

S2 Table Primer and probe sequences and amplicon lenghts and details. (XLSX)

S3 Table Reagent and consumables catalog numbers and manufacturer details. (XLSX)

S4 Table Droplet counts per cluster. (XLSX)

S5 Table Molecule copies per partition statistics. (XLSX)

S6 Table The range, mean, standard deviation (*σ*), and coefficient of variation in percent (%CV) of values represented by their *NEB* TRI CN determined by aCGH and the *NEB* TRI exon IV and exon VIII ddPCR assays. (XLSX)

S7 Table Intra-assay analysis. (XLSX)

S8 Table Inter-assay analysis. (XLSX)

S9 Table Linear regression values. (XLSX)

S10 Table Cohen’s kappa table and values for detection of copy number comparison between ddPCR and aCGH. (XLSX)

S11 Table Additional Cohen’s kappa statistics for the detection of pathogenicity comparison between ddPCR and aCGH. (XLSX)

S12 Table *NEB* TRI exon IV filtered data. (XLSX)

S13 Table *NEB* TRI exon VIII filtered data. (XLSX)

S1 Fig Example 2D droplet intensity plots of *NEB* TRI exon IV and exon VIII assays on patient DNA and no-template controls as extracted from the Quantasoft Analysis Pro software. (TIF)
