# Supplementary figures and images for: A custom ddPCR method for the detection of copy number variations in the nebulin triplicate region
Source: PLoS One. 2022 May 16;17(5):e0267793. doi: 10.1371/journal.pone.0267793 (PMC9109913; doi:10.1371/journal.pone.0267793)

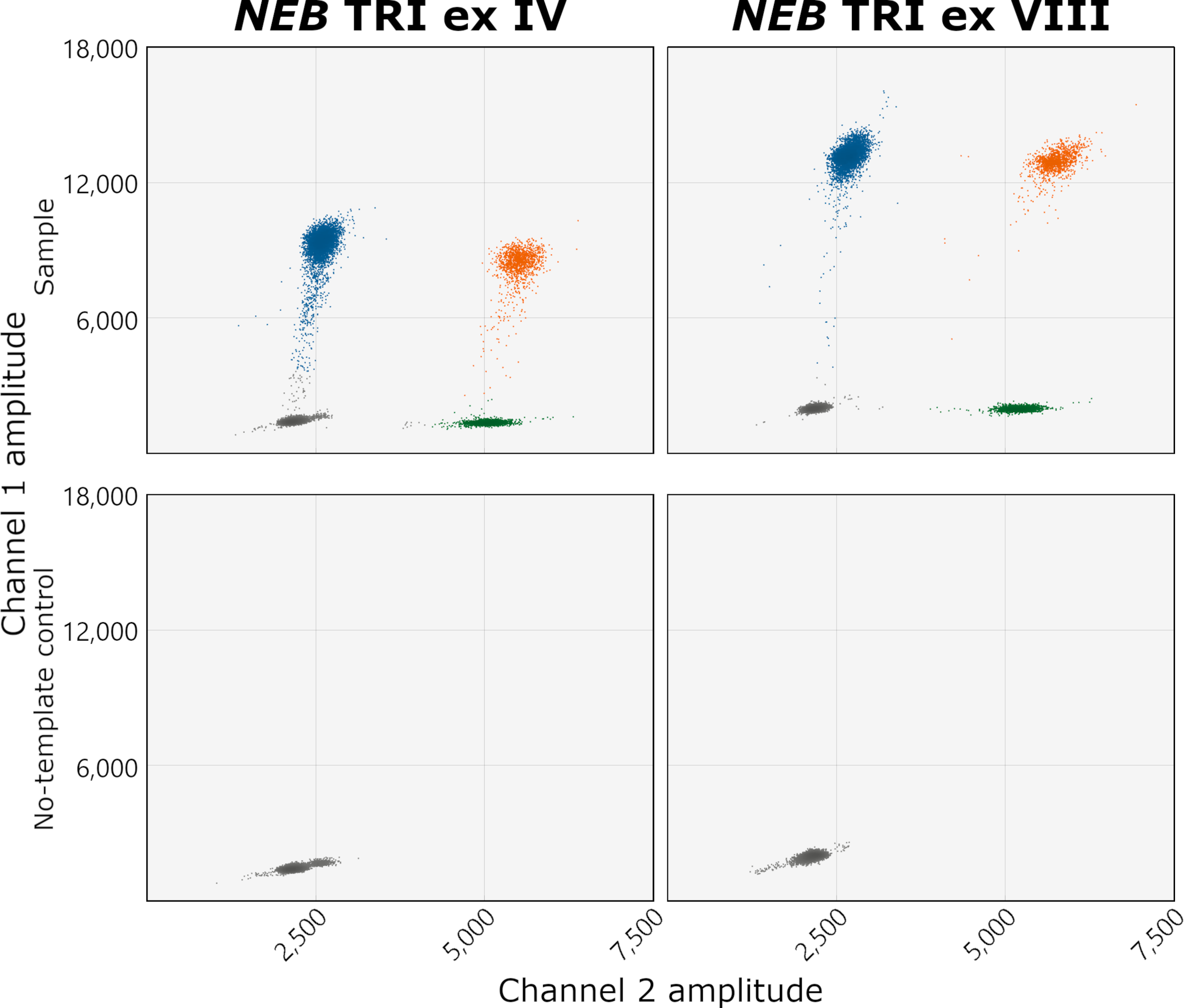

Supplement: S1 Fig — (TIF) [file pone.0267793.s014.tif]
